# Sleep quality disparities in different pregnancy trimesters in low- and middle-income countries: a systematic review and meta-analysis

**DOI:** 10.1186/s12884-024-06830-3

**Published:** 2024-10-01

**Authors:** Esuyawkal Mislu, Henok Kumsa, Shimelis Tadesse, Mulugeta Wodaje Arage, Belay Susu, Mulat Ayele, Fiker Chane

**Affiliations:** 1 School of Midwifery, College of Health Science, Woldia University, Woldia, Ethiopia; 2 Department of Midwifery, College of Health Science, Mettu University, Mettu, Ethiopia

**Keywords:** Sleep quality, Poor sleep quality, Pittsburg sleep quality index, Different trimesters, Systematic review

## Abstract

**Introduction:**

Sleep is a crucial determinant of maternal and fetal health, significantly impacting the well-being of both the mother and her developing fetus. Poor sleep quality, characterized by difficulties in falling asleep or staying asleep, can cause poor pregnancy outcome. Conversely, studies came with inconsistent result in the prevalence of poor sleep quality in different trimester of pregnancy. Therefore, this systematic review and meta-analysis study aimed to compare the prevalence of poor sleep quality in different trimesters.

**Method:**

A systematic review and meta-analysis were done on published studies. Electronic data base search was done from PubMed, Hinari, Medline and Google Scholar. Data were extracted with Excel and the analysis were done using STATA version 17. Publication bias was assessed both graphically and statistically. I-square test was used to identify heterogeneity.

**Result:**

In this meta-analysis, 38 studies that measured poor sleep quality using the Pittsburg Sleep Quality Index (PSQI ≥ 5) were included. The pooled prevalence of poor sleep quality was identified as 37.46% (95% CI: 29.26, 45.67) in the first trimester, 47.62% (95% CI: 42.23, 53.02) in the second trimester, and 60.05% (95% CI: 51.32, 68.78) in the third trimester.

**Conclusion:**

This study identified a significant discrepancy in the prevalence of poor sleep quality, which increases as gestational age advances. Therefore, this discrepancy should be addressed, and additional support should be provided to pregnant women to help them achieve adequate sleep, especially as gestational age advances.

**Supplementary Information:**

The online version contains supplementary material available at 10.1186/s12884-024-06830-3.

## Introduction

Sleep is crucial for the growth and development of the fetus and is integral to a positive pregnancy experience [1]. Adequate sleep ensures the baby receives necessary resources for optimal growth [1, 2]. However, many pregnant women face challenges with sleep quality, including difficulty falling asleep, staying asleep, or achieving restful sleep [3].

Several factors can affect sleep quality during pregnancy, such as hormonal changes, physical discomfort (e.g., nausea, breast tenderness), increased urination frequency, and emotional factors like anxiety, and worries about the baby’s health [4, 5]. Poor sleep quality during pregnancy has been linked to adverse outcomes such as a higher risk of gestational diabetes, preeclampsia, preterm birth, low birth weight, and postpartum depression [6–8]. It also negatively impacts overall well-being, leading to daytime fatigue, reduced cognitive function, and difficulty coping with the physical and emotional demands of pregnancy [3, 6, 9].

The quality of sleep varies significantly across trimesters. In the first trimester, hormonal changes and physical discomfort can hinder restful sleep [10]. Moving in to the second trimester, challenges include increased urination and difficulty finding a comfortable sleeping position due to the growing abdomen, alongside emotional factors like anxiety [11]. By the third trimester, physical discomfort intensifies with symptoms such as back pain and difficulty breathing, compounded by concerns about labor, delivery, and preparing for the baby’s arrival [4, 5].

Addressing poor sleep quality is crucial, and it requires awareness from pregnant women, healthcare providers, and support systems [6, 12, 13]. Strategies like maintaining good sleep hygiene, practicing relaxation techniques before bed, creating a comfortable sleep environment, and seeking medical advice for sleep disturbances can help improve sleep quality and mitigate its negative consequences during pregnancy [14–16].

Despite recognizing the issue, studies have shown a wide range in the reported prevalence of poor sleep quality in different trimester of pregnancy. Therefore, this systematic review and meta-analysis was conducted to compare the prevalence of poor sleep quality in different trimester of pregnancy in low- and middle-income countries which is crucial better understand these variations.

## Methods

This systematic review and meta-analysis was conducted to determine the prevalence of poor quality of sleep and compare it among different trimester pregnancy in low- and middle-income countries. The review strictly followed the Preferred Reporting Items for Systematic reviews and Meta-Analysis (PRISMA 2020) guidelines [17].

### Search strategy

Electronic search of online databases was done from PubMed, Medline, Google Scholar, and Hinari databases to retrieve published articles conducted on the prevalence of poor quality of sleep among pregnant women. In addition, we extended our search by retrieving and extracting potential articles from reference lists of eligible articles. The searching was conducted using free text and Medical Subject Heading (MeSH) terms such as: sleep, sleep quality, poor sleep quality, prevalence, magnitude, Pittsburgh Sleep Quality Index, PSQI, and pregnancy.

### Inclusion criteria

All articles of a cross-sectional, retrospective cohort, and prospective cohort studies that assess the prevalence of poor quality of sleep among different trimester of pregnancy in low and middle-income countries and written in English were included. Agreements on the inclusion and exclusion of the articles were done through extensive discussion of all authors.

### Exclusion criteria

Studies were excluded if they were case reports, case series, conference papers, reviews, or published only as abstracts. We also excluded studies that did not use the PSQI score to measure sleep quality, those that did not report prevalence data on poor sleep quality, and studies involving non-pregnant women. Duplicate publications were also excluded.

### Outcome measurement

The included studies measured poor sleep quality by the Pittsburg Sleep Quality Index (PSQI), a PSQI score ≥ 5.

### Data extraction and quality assessment

The Joana Brigg’s Institute (JBI) tool for prevalence and determinant studies was used as a guideline for data extraction from the final selected articles. The data were extracted in Microsoft excel form, checked and evaluated by all authors. The extraction sheet contains name of authors, study year, publication year, country and region of study, study design, study population, sample size, prevalence with the upper and lower boundary confidence intervals. The quality of included studies was evaluated using the Joanna Briggs Institute (JBI) critical appraisal checklist [18]. The quality scores of included studies were assessed and presented using the mean scores to designate them as high- or low-quality. Articles with a score of 5 or more out of 8 were considered to be of good quality and were included in the review. Low- and middle-income countries list were identified from World Bank official website (countries with 2022 GNI per capita was less than $13,845) [19].

### Data processing and analysis

The extracted data were analyzed using STATA 17. The results were presented in evidence tables, figures and descriptive statistics form. The estimates for prevalence from each study were pooled and determined as a single estimate with its corresponding 95% CI for each trimester of pregnancy. The random effect regression model was used for analysis [20]. Forest plots were used to estimate the pooled prevalence and effect size of each study with their 95% confidence interval. The size of each box indicated the weight of the study, while each crossed line refers to the 95% confidence interval. Subgroup analysis was done by sub-region and income level of country’s the study were from, which enables the assessment of poor sleep quality varies in different low- and middle-income countries.

### Heterogeneity and publication biases assessment

The magnitudes of heterogeneity between studies were assessed using I^2^ statistics and values of 25%, 50%, and 75% were graded as low, medium, and high quality respectively. A P-value of less than 0.1 was considered to suggest statistically significant heterogeneity [21]. For further clarification on the source of heterogeneity, the pooled prevalence was reported on the base of income level by subgroup analysis. Publication bias was assessed subjectively by observing the funnel plot and objectively by considering Egger’s test estimates at a 5% level of significance [22, 23]. Leave-one-out sensitivity analysis was performed to confirm potentially biased direction of pooled estimates on Egger’s test [24].

### Registration and protocol

This review has been previously registered on PROSPERO (ID: CRD42024496841).

## Result

### Study selection

**Fig. 1:**
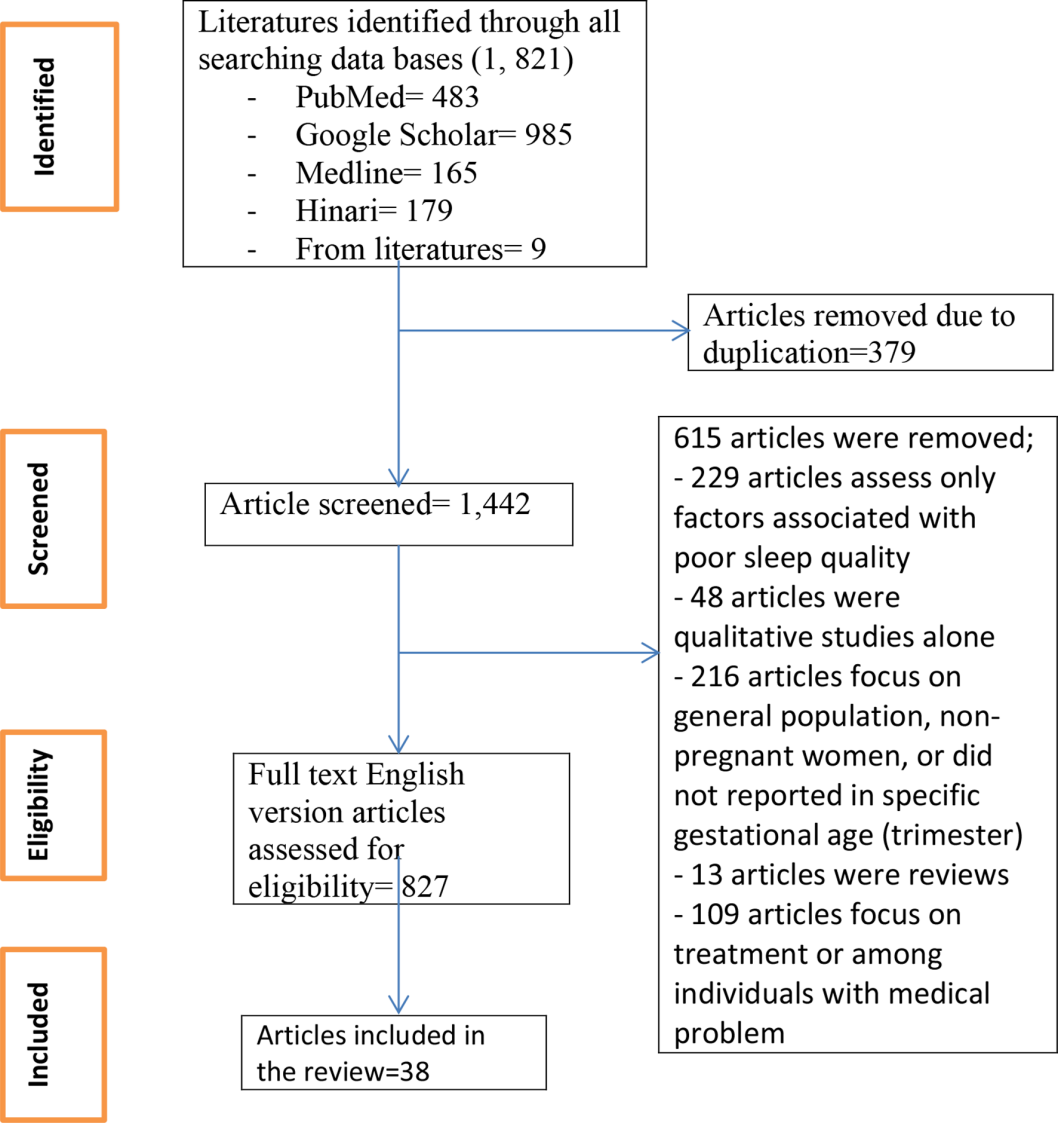
Flow chart for studies selection to compare the pooled prevalence of poor sleep quality in different trimester of pregnancy in low- and middle-income countries

This systematic review and meta-analysis included published studies on the prevalence of poor sleep quality among pregnant women in different trimester in low and middle-income countries. More than 1,821 articles were identified through electronic database searching, and 38 studies were included to estimate and compare the pooled prevalence of poor sleep quality in different trimester of pregnancy (Fig. 1).

### Characteristics of included studies

This systematic review and meta-analysis included 38 studies from 13 different low- and middle-income countries without restriction based on the year of publication. Eleven studies from China [13, 25–34], six studies from Indonesia [35–40], five studies from Ethiopia [41–45], two studies from Peru [46, 47], three studies from Iran [48–50], two studies from Pakistan [51, 52], two studies from India [53, 54], two studies from Turkey [55, 56], and five studies from Malaysia [12], Russia [57], Sri Lanka [58], Brazil [59], and Thailand [60], one from each countries. In this meta-analysis, 11,610, 7168 and 9265 women were included in the first, second and third trimester of pregnancy, respectively.

Based on income categories of countries, as World Bank categorized them based on their income, five studies were from low-income countries [41–45], eight studies from lower middle-income countries [48–54, 58], and twenty-five studies from upper middle-income countries [12, 13, 25–40, 46, 47, 55–57, 59, 60] (Supplementary Table 1).

### Risk of bias assessment

Publication bias was comprehensively assessed through both graphical and statistical methods in this review. Funnel plots were presented for the first (Supplementary Fig. 1), second (Supplementary Fig. 2), and third trimesters (Supplementary Fig. 3), visually inspecting the distribution of study outcomes. Additionally, Egger’s tests were conducted, yielding p-values of 1.00 for the first trimester and second trimester, and 0.9148 for the third trimester, indicating no significant evidence of publication bias across these trimesters in the meta-analysis.

### Prevalence of poor quality of sleep in the first trimester of pregnancy

There were 17 studies reporting on the prevalence of poor sleep quality during the first trimester of pregnancy. The pooled prevalence of poor sleep quality among pregnant women during this period was 37.46% (95% CI: 29.26, 45.67). However, the I-square statistic (I² = 98.8%) showed a significant heterogeneity among the included studies.

**Fig. 2:**
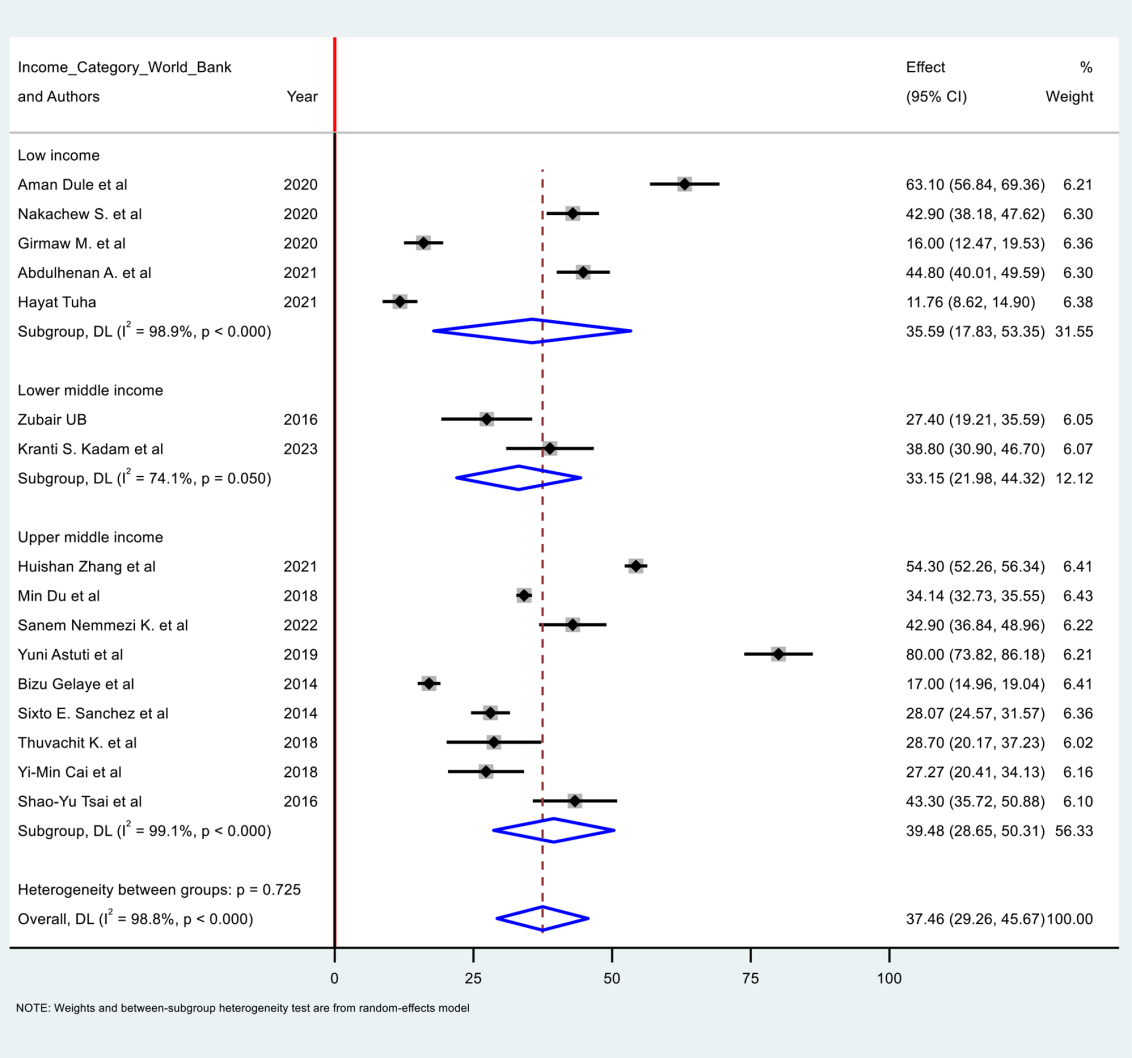
Forest plot for poor sleep quality among first trimester pregnant women in low- and middle-income countries

Subgroup analysis was conducted based on income categories. The result revealed a prevalence of 35.59% (95% CI: 17.83, 35.35) in low-income countries, 33.15% (95% CI: 21.98, 44.32) in lower-middle-income countries, and 39.48% (95% CI: 28.69, 50.31) in upper-middle-income countries (Fig. 2).

### Prevalence of poor sleep quality in the second trimester of pregnancy

**Fig. 3:**
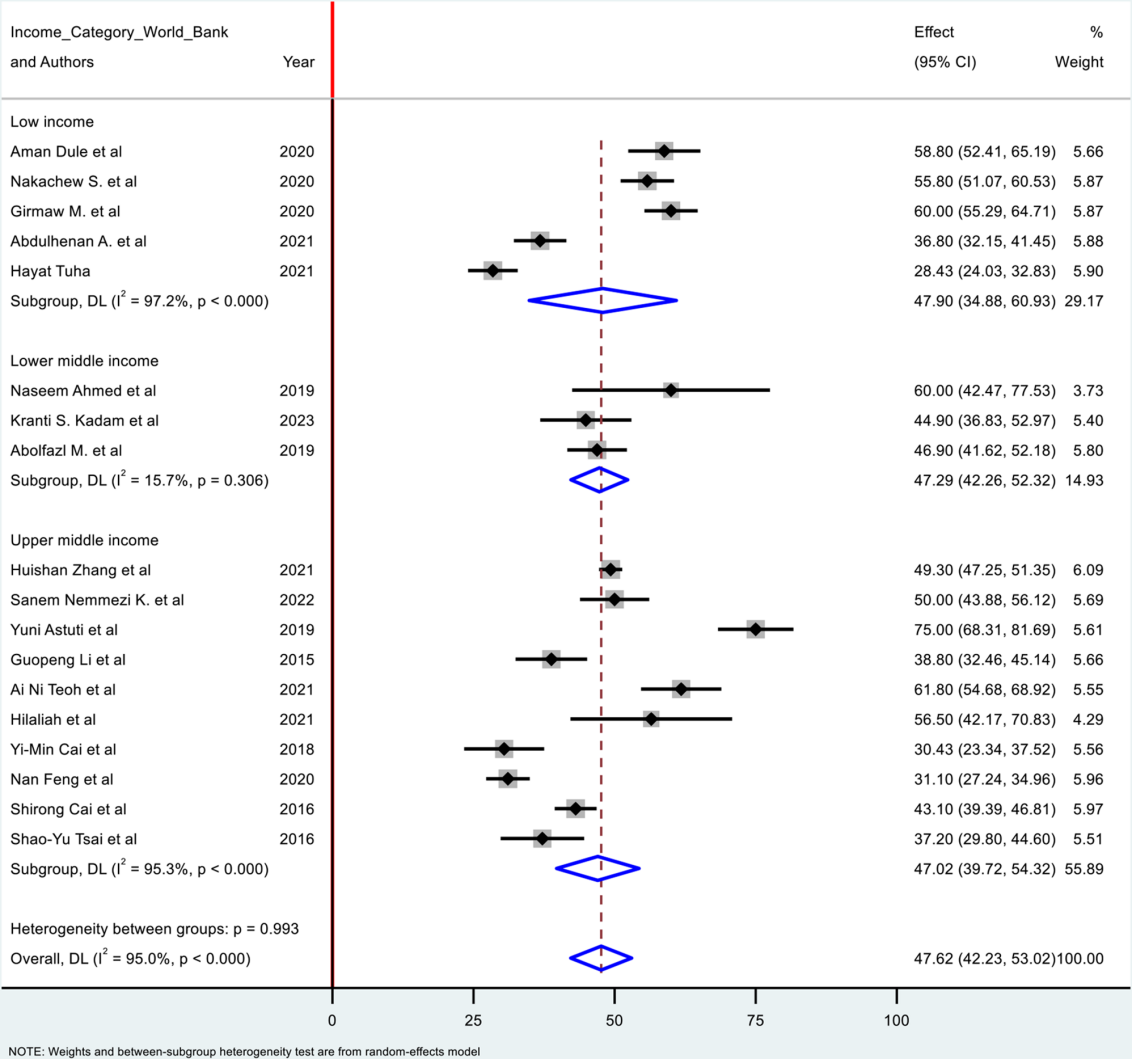
Forest plot for poor quality of sleep among pregnant women during the second trimester of pregnancy in low and middle-income countries

There were 18 studies reported the prevalence of poor sleep quality among pregnant women during the second trimester of pregnancy. The pooled prevalence of poor sleep quality during the second trimester of pregnancy were 47.623% (95% CI: 42.230, 53.016). However, there was high degree of heterogeneity among the studies identified from the I² (I^2^ = 95.0%), for which sub group analysis was done based on income categories. The pooled prevalence were 47.90% (95% CI: 34.88, 60.93) in low income countries, 47.29% (95% CI: 42.26, 52.32) in lower middle-income countries, and 47.02% (95% CI: 39.72, 54.32) in upper middle-income countries (Fig. 3).

### Prevalence of poor quality of sleep during the third trimester of pregnancy

**Fig. 4:**
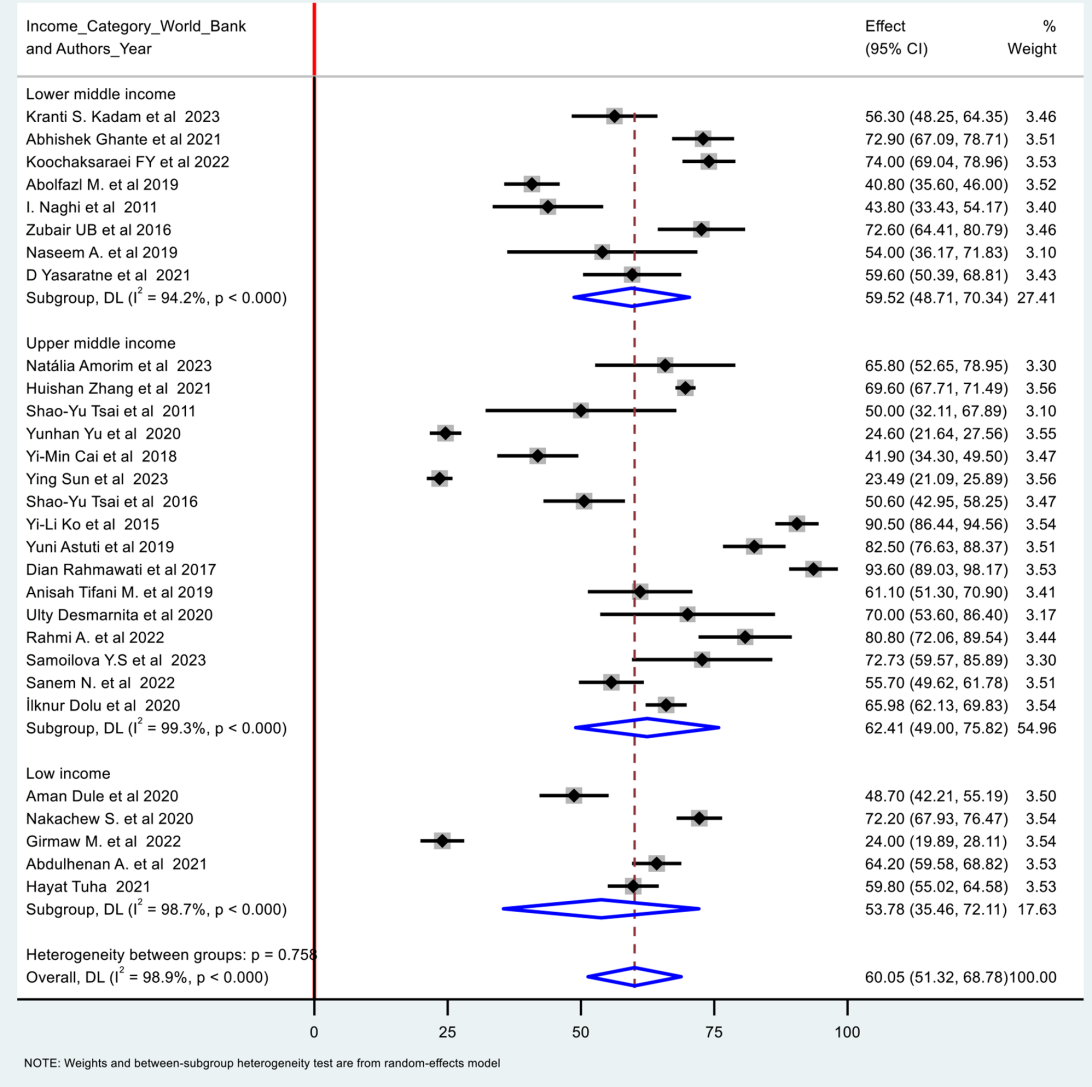
Forest plot for poor quality of sleep during the third trimester of pregnancy in low and middle-income countries

There were 28 studies reported the prevalence of poor sleep quality during the third trimester of pregnancy. The pooled prevalence was identified as 60.05% (95% CI: 48.700, 66.623). I-square result (I² (%): 98.9%) showed a significant heterogeneity among the included studies. The pooled prevalence were 53.78% (95% CI: 35.46, 72.11) in low income countries, 59.52% (95% CI: 48.71, 70.34) in lower middle-income countries, and 62.41% (95% CI: 49.00, 75.82) in upper middle-income countries (Fig. 4). Summary of the statistics and meta-analysis is shown on Table 1.

**Table 1:** Summary table to compare the prevalence of poor sleep quality in different trimester of pregnancy in low- and middle-income countries

Women’s characteristics	Included studies	Number of participant	Pooled prevalence (%)	95% CI	I-square (%)	Publication bias tests
First trimester	17	11,610	37.46	29.26, 45.67	98.8	Egger test (*p* = 1.0000)
*Subgroup analysis based on income level*						
LIC	5	1884	35.59	17.83, 35.35	98.9	
LMIC	2	260	33.15	21.98, 44.32	74.1	
UMIC	10	9466	39.48	28.69, 50.31	99.1	
Second trimester	18	7168	47.62	42.23, 53.02	95.0	Egger test (*p* = 1.0000)
*Subgroup analysis based on income level*						
LIC	5	1884	47.90	34.88, 60.93	97.2	
LMIC	3	519	47.29	42.26, 52.32	15.7*	
UMIC	10	4538	47.02	39.72, 54.32	95.3	
Third trimester	28	9265	60.05	51.32, 68.78	98.9	Egger test (*p* = 0.9148)
*Subgroup analysis based on income level*						
LIC	5	1884	53.78	35.46, 72.11	98.7	
LMIC	7	1129	59.52	48.71, 70.34	94.2	
UMIC	16	6252	62.41	49.00, 75.82	99.3	

LIC: low income countries; LMIC: lower middle-income countries; UMIC: upper middle income countries; * low heterogeneity

## Discussion

This systematic review and meta-analysis highlights a marked increase in the prevalence of poor sleep quality among pregnant women as pregnancy progresses, with rates escalating from 37.46% in the first trimester to 47.62% in the second trimester, and reaching 60.05% in the third trimester. This escalation is consistent with previous reviews conducted by Salari Nader et al. [11] and Ivan D. Sedov et al. [61], which also reported an increasing prevalence of poor sleep quality during the latter stages of pregnancy. Moreover, studies conducted in upper-income countries, including the USA [62], Poland [63], and Saudi Arabia [64] supports this finding, suggesting that the observed escalation is a widespread phenomenon. The main cause of this significant deterioration of sleep quality is the physiological, social and psychological changes accompanying pregnancy [65].

The marked increase in poor sleep quality across trimesters suggests that pregnant women encounter progressively more complex and distinct challenges to their sleep as their pregnancy advances. Several factors might contribute to these discrepancies: hormonal changes, particularly the fluctuations in progesterone and estrogen, disrupt sleep patterns by affecting thermoregulation and increasing nighttime awakenings [63, 66, 67]. Physical discomfort also intensifies, with the growing uterus applying pressure on internal organs and the bladder, leading to frequent urination and discomfort that interrupts sleep. Additionally, increased fetal movement, especially in the third trimester, can be disruptive, making it harder for women to fall and stay asleep [63, 67, 68].

Gastrointestinal issues, such as acid reflux, heartburn, indigestion, and constipation, become more prevalent as the pregnancy advances due to the expanding uterus exerting pressure on the stomach and intestines [11, 69, 70]. The progressively increasing leg cramps and restless legs syndrome is another common issue in the later stages of pregnancy, contributing to nocturnal discomfort [71]. Psychological factors, including heightened anxiety and stress related to labor, delivery, and parenting responsibilities, further exacerbate increase as pregnancy advances. These concerns can exacerbate sleep disturbances, as women worry about potential complications or the health of their baby [72, 73].

The review also included a subgroup analysis based on the income level of the countries where studies were conducted, examining low-income, lower-middle-income, and upper-middle-income settings. The results indicated no statistically significant differences in the prevalence of poor sleep quality across these income levels, suggesting that poor sleep quality during pregnancy is a universal issue affecting women regardless of economic status [72, 74].

The findings indicate that poor sleep quality among pregnant women worsens as gestational age progresses, underscoring the need for increased support from healthcare providers and family members. This decline in sleep quality, particularly during late pregnancy, may exacerbate stress, anxiety, and mood disorders, highlighting the necessity for targeted interventions [72]. Healthcare providers should implement comprehensive care strategies, including education on sleep hygiene, routine screening for sleep disorders, and psychological support, to enhance sleep quality and overall well-being throughout pregnancy [75]. Addressing these issues proactively is essential for improving outcomes for pregnant women as they advance through their pregnancy [65].

While interpreting the findings of this review, it is better to consider the following strengths and limitations. As a strength, this review included only studies that measure the quality of sleep using similar measurement tool (the Pittsburgh Sleep Quality Index). As a limitation, this study only included published studies and those written in the English language, and there was significant heterogeneity among the included studies.

## Conclusion

This review identified a considerable heterogeneity among the included studies and a significant discrepancy in the prevalence of poor sleep quality across different trimesters of pregnancy, with a trend showing an increase in poor sleep quality as gestational age advances. This observation suggests that the sleep quality of pregnant women deteriorate as their pregnancies progress. Interventions aimed at improving sleep hygiene, providing supportive prenatal care, and addressing specific physical discomforts associated with pregnancy could potentially mitigate these challenges and improve maternal and fetal health outcomes.

Further meta-analysis studies also needed to explore factors contributing to poor sleep quality among pregnant women, and interventions to improve sleep quality for pregnant women.

## Electronic supplementary material

Below is the link to the electronic supplementary material.

Supplementary Material 1

Supplementary Material 2

## Data Availability

The datasets used in this study are available from the authors upon reasonable request.

## Abbreviations

CI: Confidence interval
PSQI: Pittsburg Sleep Quality Index

## References

[1] Gupta R, Rawat VS. Chapter 10 - sleep and sleep disorders in pregnancy. In: Steegers EAP, Cipolla MJ, Miller EC, editors. Handbook of clinical neurology. Volume 172. Elsevier; 2020. pp. 169–86. 10.1016/B978-0-444-64240-0.00010-6 32768087

[2] Liu H, Li H, Li C, Chen L, Zhang C, Liu Z, et al. Associations between maternal sleep quality throughout pregnancy and newborn birth weight. Behav Sleep Med. 2021;19(1):57–69. 31830816 10.1080/15402002.2019.1702551

[3] Toffol E, Kalleinen N, Urrila AS, Himanen S-L, Porkka-Heiskanen T, Partonen T et al. The relationship between mood and sleep in different female reproductive states. BMC Psychiatry. 2014;14(1):177.10.1186/1471-244X-14-177. 10.1186/1471-244X-14-177 PMC4071019 24935559

[4] Conlon RPK, Wang B, Germeroth LJ, Cheng Y, Buysse DJ, Levine MD. Demographic, pregnancy-related, and health-related factors in association with changes in sleep among pregnant women with overweight or obesity. Int J Behav Med. 2021;28:200–6. 32378048 10.1007/s12529-020-09887-4 PMC12990154

[5] Smyka M, Kosińska-Kaczyńska K, Sochacki-Wojcicka N, Zgliczyńska M, Wielgoś M. Sleep quality according to the Pittsburgh Sleep Quality Index in over 7000 pregnant women in Poland. Sleep Biol Rhythms. 2021;19(4):353–60.

[6] Lu Q, Zhang X, Wang Y, Li J, Xu Y, Song X, et al. Sleep disturbances during pregnancy and adverse maternal and fetal outcomes: a systematic review and meta-analysis. Sleep Med Rev. 2021;58:101436. 33571887 10.1016/j.smrv.2021.101436

[7] Johns EC, Denison FC, Reynolds RM. Sleep disordered breathing in pregnancy: a review of the pathophysiology of adverse pregnancy outcomes. Acta Physiol. 2020;229(2):e13458. 10.1111/apha.13458 32087033

[8] Zhou H, Li W, Ren Y. Poor sleep quality of third trimester exacerbates the risk of experiencing postnatal depression. Psychol Health Med. 2020;25(2):229–38. 30450954 10.1080/13548506.2018.1549738

[9] Pauley AM, Moore GA, Mama SK, Molenaar P, Symons Downs D. Associations between prenatal sleep and psychological health: a systematic review. J Clin Sleep Med. 2020;16(4):619–30. 32003734 10.5664/jcsm.8248 PMC7161464

[10] Yang Y, Mao J, Ye Z, Zeng X, Zhao H, Liu Y, et al. Determinants of sleep quality among pregnant women in China: a cross-sectional survey. J Maternal-Fetal Neonatal Med. 2018;31(22):2980–5. 10.1080/14767058.2017.1359831. 10.1080/14767058.2017.1359831 28738757

[11] Salari N, Darvishi N, Khaledi-Paveh B, Vaisi-Raygani A, Jalali R, Daneshkhah A, et al. A systematic review and meta-analysis of prevalence of insomnia in the third trimester of pregnancy. BMC Pregnancy Childbirth. 2021;21:1–8. 33836686 10.1186/s12884-021-03755-z PMC8034118

[12] Teoh AN, Kaur S, Mohd Shukri NH, Shafie SR, Ahmad Bustami N, Takahashi M, et al. Psychological state during pregnancy is associated with sleep quality: preliminary findings from MY-CARE cohort study. Chronobiol Int. 2021;38(7):959–70. 33779445 10.1080/07420528.2021.1902338

[13] Du M, Liu J, Han N, Zhao Z, Yang J, Xu X, et al. Maternal sleep quality during early pregnancy, risk factors and its impact on pregnancy outcomes: a prospective cohort study. Sleep Med. 2021;79:11–8. 33454523 10.1016/j.sleep.2020.12.040

[14] Baglioni C, Tang NK, Johann AF, Altena E, Bramante A, Riemann D, et al. Insomnia and poor sleep quality during peripartum: a family issue with potential long term consequences on mental health. J Maternal-Fetal Neonatal Med. 2022;35(23):4534–42. 10.1080/14767058.2020.1854718 33267621

[15] Facco FL, Chan M, Patel SR. Common sleep disorders in pregnancy. Obstet Gynecol. 2022;140(2):321–39. 35852285 10.1097/AOG.0000000000004866

[16] Oats JJ, Boyle J. Llewellyn-Jones fundamentals of Obstetrics and Gynaecology. E-Book: Elsevier Health Sciences; 2022.

[17] Page MJ, Moher D, McKenzie JE. Introduction to PRISMA 2020 and implications for research synthesis methodologists. Res Synthesis Methods. 2022;13(2):156–63. 10.1002/jrsm.1535 34693644

[18] Munn Z, Moola S, Lisy K, Riitano D, Tufanaru C. Systematic reviews of prevalence and incidence. Joanna Briggs Institute reviewer’s manual Adelaide, South Australia: The Joanna Briggs Institute. 2017:5.1-5.

[19] Bank W. Low & middle income: World Bank; 2024 [ https://data.worldbank.org/income-level/low-and-middle-income

[20] Borenstein M, Hedges LV, Higgins JP, Rothstein HR. A basic introduction to fixed-effect and random-effects models for meta-analysis. Research synthesis methods. 2010;1(2):97-111.10.1002/jrsm.12. 10.1002/jrsm.12 26061376

[21] Higgins J, Thompson S, Deeks J, Altman D. Measuring inconsistency in meta-analyses BMJ. 2003; 327: 557–60. Journal of Thoracic Disease All rights reserved http://dx doi org/1021037/jtd-20-3130 Supplementary© Journal of Thoracic Disease All rights reserved http://dx doi org/1021037/jtd-20-3130. 10.1136/bmj.327.7414.557 PMC192859 12958120

[22] Egger M, Davey Smith G, Schneider M, Minder C. Bias in meta-analysis detected by a simple, graphical test. BMJ. 1997;315(7109):629 – 34.10.1136/bmj.315.7109.629. 10.1136/bmj.315.7109.629 PMC2127453 9310563

[23] Begg CB, Mazumdar M. Operating characteristics of a rank correlation test for publication bias. Biometrics. 1994:1088 – 101. 7786990

[24] Duval S, Tweedie R. A nonparametric trim and fill method of accounting for publication bias in meta-analysis. J Am Stat Assoc. 2000;95(449):89–98.

[25] Zhang H, Li P, Fan D, Wu S, Rao J, Lin D et al. Prevalence of and risk factors for poor sleep during different trimesters of pregnancy among women in China: a cross-sectional study. Nat Sci Sleep. 2021:811–20. 10.2147/NSS.S303763 PMC8216751 34168511

[26] Cai Y-M, Zheng X-L, Shen Z-M, Zhou B-F, Liu Y-M, Yang J-Y, et al. Study on the sleep quality of women pregnant with a second child and the influencing factors. Eur J Med Res. 2022;27(1):1–6. 36253870 10.1186/s40001-022-00848-z PMC9578186

[27] Tsai S-Y, Lee P-L, Lin J-W, Lee C-N. Cross-sectional and longitudinal associations between sleep and health-related quality of life in pregnant women: a prospective observational study. Int J Nurs Stud. 2016;56:45–53. 26803171 10.1016/j.ijnurstu.2016.01.001

[28] Li G, Kong L, Zhou H, Kang X, Fang Y, Li P. Relationship between prenatal maternal stress and sleep quality in Chinese pregnant women: the mediation effect of resilience. Sleep Med. 2016;25:8–12. 27823722 10.1016/j.sleep.2016.02.015

[29] Feng N, Huang X. Prevalence of depression, anxiety, sleep disorders and influencing factors among pregnant women in the second trimester in urban areas of Guangzhou city, Guangdong Province, China: a cross-sectional study. Am J Clin Exp Obstet Gynecol. 2021;7(2):21–31.

[30] Cai S, Phua DY, Tham EK, Goh DY, Teoh OH, Shek LP, et al. Mid-pregnancy and postpartum maternal mental health and infant sleep in the first year of life. J Sleep Res. 2023;32(3):e13804. 36511597 10.1111/jsr.13804

[31] Tsai S-Y, Kuo L-T, Lai Y-H, Lee C-N. Factors associated with sleep quality in pregnant women: a prospective observational study. Nurs Res. 2011;60(6):405–12. 22048560 10.1097/NNR.0b013e3182346249

[32] Yu Y, Zhu X, Xu H, Hu Z, Zhou W, Zheng B et al. Prevalence of depression symptoms and its influencing factors among pregnant women in late pregnancy in urban areas of Hengyang City, Hunan Province, China: a cross-sectional study. BMJ open. 2020;10(9). 10.1136/bmjopen-2020-038511 PMC7467533 32873680

[33] Sun Y, He X, Gu X, Yang X. Risk factors of positive depression screening during the third trimester of pregnancy in a Chinese tertiary hospital: a cross-sectional study. BMC Psychiatry. 2023;23(1):824. 37946162 10.1186/s12888-023-05343-1 PMC10636937

[34] Ko YL, Lin PC, Chen SC. Stress, sleep quality and unplanned C aesarean section in pregnant women. Int J Nurs Pract. 2015;21(5):454–61. 24754483 10.1111/ijn.12267

[35] Astuti Y, Afsah Y, editors. The Factors Influencing Sleep Quality of Pregnant Women in Yogyakarta, Indonesia. Third International Conference on Sustainable Innovation 2019–Health Science and Nursing (IcoSIHSN 2019); 2019: Atlantis Press.

[36] Arif A, Anggraini H. Relationship of obesity, anxiety level and sleep quality in pregnant women with Preeclamsia in Rsud Dr. Hm Rabain Muara Enim 2021. Sci Midwifery. 2022;10(2):829–34.

[37] Rahmawati D, Ungsianik T, editors. Anxiety, depression, and sleep quality of third trimester pregnant women. International Nursing Student Symposium and Festival; 2017.

[38] Maulidyanti AT, Widiyanti D, Heryati K, editors The Relationship Between Sleep Quality And Depression During The Third Trimester Of Pregnant Women In The Public Health Center In Bengkulu City. 1st International Conference on Inter-Professional Health Collaboration (ICIHC 2018). 2019: Atlantis Press.

[39] Desmarnita U, Fadilasari Y, Mulyanti Y. Discomfort of pregnant women on the quality sleep of third trimester pregnant women. Asian J Pharm Res Dev. 2023;11(4):7–11.

[40] Yuda RA, Yanis A, Karmia HR. The Association of Maternal Sleep Quality with Newborn Health. Sci Midwifery. 2022;10(5):4370–7.

[41] Dule A, Abdu Z, Hajure M, Mohammedhussein M. Sleep quality among pregnant women amidst COVID-19: Association with Mental Wellbeing and Self-efficacy. Am J Health Res. 2021;9(6):238–45.

[42] Amare NS, Chekol B, Aemro A. Determinants of Poor Sleep Quality During the COVID-19 Pandemic Among Women Attending Antenatal Care Services at the Health Facilities of Debre Berhan Town, Ethiopia: An Institutional-Based Cross-Sectional Study. Frontiers in Psychiatry. 2022;13.10.3389/fpsyt.2022.841097. 10.3389/fpsyt.2022.841097 PMC8971521 35370833

[43] Takelle GM, Muluneh NY, Biresaw MS. Sleep quality and associated factors among pregnant women attending antenatal care unit at Gondar, Ethiopia: a cross-sectional study. BMJ open. 2022;12(9):e056564.10.1136/bmjopen-2021-056564. 10.1136/bmjopen-2021-056564 PMC9445783 36691143

[44] Abdurahman A, Dagnew B, Yismaw Gela Y, Akalu Y, Ashenaf Yibeyine B, Diress M et al. Sleep Quality and Associated Factors among pregnant women attending Antenatal Care Unit at the Referral hospitals in Oromia National Regional State, Ethiopia, 2021: a Multicenter cross-sectional study. Behav Sleep Med. 2023:1–15. 10.1080/15402002.2023.2232499 37461301

[45] Tuha H. Quality of Sleep and Associated Factors among pregnant women in AddisAbaba. Public Hospitals: uog; 2021.

[46] Gelaye B, Addae G, Neway B, Larrabure-Torrealva GT, Qiu C, Stoner L, et al. Poor sleep quality, antepartum depression and suicidal ideation among pregnant women. J Affect Disord. 2017;209:195–200. 10.1016/j.jad.2016.11.020. 27930912 10.1016/j.jad.2016.11.020 PMC5360461

[47] Sanchez SE, Islam S, Zhong Q-Y, Gelaye B, Williams MA. Intimate partner violence is associated with stress-related sleep disturbance and poor sleep quality during early pregnancy. PLoS ONE. 2016;11(3):e0152199. 27023005 10.1371/journal.pone.0152199 PMC4811406

[48] Mehdizadehkashi A, Mobasseri A, Chaichian S, Tahermanesh K, Haghighi L, Hashemi N, et al. The frequency of pregnancy Associated Sleep disorders among pregnant mothers who referred to Rasoul-Akram hospital for prenatal care 2018–2019. Shiraz E-Med J. 2020;22(8):e106883. 10.5812/semj.106883.

[49] Koochaksaraei FY, Nasiri-Amiri F, Faramarzi M, Khafari S, Kheirkhah F. Late-pregnancy sleep quality and psychological distress in Iranian primiparous women. Casp J Reproductive Med. 2022;8(1):1–8.

[50] Naghi I, Keypour F, Ahari S, Tavalai S, Khak M. Sleep disturbance in late pregnancy and type and duration of labour. J Obstet Gynaecol. 2011;31(6):489–91. 21823845 10.3109/01443615.2011.579196

[51] Zubair U, Malik Z, Ali U. Assessment of Quality of Sleep in Pregnancy and Socio Demographic Factors Associated with Poor Sleep Quality in Pregnancy. J Sleep Disor: Treat Care 5: 2. of. 2016;4:2.

[52] Ahmed N, Khan A, Waseem A, Khan T, Shabbir H, Waqas A. Prevalence of sleep disturbances during pregnancy–a pilot study. BAOJ Gynaec. 2019;2(3):1–8.

[53] Kadam KS, Anvekar AR, Unnithan VB. Depression, sleep quality, and body image disturbances among pregnant women in India: a cross-sectional study. J Yeungnam Med Sci. 2023;40(4):394. 37157779 10.12701/jyms.2023.00087 PMC10626296

[54] Ghante A, Raj JP, Krishna B, Thomas A. Prevalence and predictors of sleep deprivation and poor sleep quality and their associated perinatal outcomes during the third trimester of pregnancy. J Taibah Univ Med Sci. 2021;16(3):359–64. 34140862 10.1016/j.jtumed.2020.11.008 PMC8178615

[55] Karaca SN, YILDIZ Ç. Evaluation of sleep quality and related factors in pregnant women. Cumhuriyet Med J. 2022;44(3):296–303.

[56] Dolua İ, Pirob TJ, Bilgilic N, Safarid K, Eryüze E, Mirkhan H. Factors affecting subjective sleep quality in the third trimester of pregnancy in urban areas of Turkey and Iraq. Revista Argentina De Clínica Psicológica. 2020;29(5):1750–65.

[57] Samoilova YS, Reutskaya VV. Screening for sleep apnea, daytime sleepiness, sleep quality, anxiety and depressive disorders in pregnant women in the third trimester of pregnancy. Молодежный инновационный вестник. 2023;12:41–4.

[58] Yasaratne D, Deen A, Anuththara T, De Silva D, Ratnayake C, Kandauda C et al. Factors associated with poor sleep quality and excessive daytime sleepiness in late pregnancy: a pilot study in an antenatal unit. Sri Lanka J Obstet Gynecol. 2022;44(1).

[59] Felix NAR, Ceolim MF. Sleep in pregnancy quarters: a longitudinal study. Revista Gaúcha De Enfermagem. 2022;44. 10.1590/1983-1447.2023.20210278.en 36541948

[60] Kriengtuntiwong T, Zaw YH, Taneepanichskul N. Brain-derived neurotrophic factor (BDNF) depression and subjective sleep quality in the first trimester of pregnancy among migrant workers in Thailand. J Multidisciplinary Healthc. 2021:2549–56. 10.2147/JMDH.S322355 PMC8450157 34552333

[61] Sedov ID, Cameron EE, Madigan S, Tomfohr-Madsen LM. Sleep quality during pregnancy: a meta-analysis. Sleep Med Rev. 2018;38:168–76. 10.1016/j.smrv.2017.06.005. 28866020 10.1016/j.smrv.2017.06.005

[62] Facco FL, Kramer J, Ho KH, Zee PC, Grobman WA. Sleep Disturbances in Pregnancy. Obstetrics & Gynecology. 2010;115(1):77-83.10.1097/AOG.0b013e3181c4f8ec. 10.1097/AOG.0b013e3181c4f8ec 20027038

[63] Smyka M, Kosińska-Kaczyńska K, Sochacki-Wójcicka N, Zgliczyńska M, Wielgoś M. Sleep problems in Pregnancy—A cross-sectional study in over 7000 pregnant women in Poland. Int J Environ Res Public Health. 2020;17(15):5306. https://www.mdpi.com/1660-4601/17/15/5306. 32717974 10.3390/ijerph17155306 PMC7432323

[64] Al-Musharaf S. Changes in sleep patterns during pregnancy and predictive factors: a longitudinal study in Saudi Women. Nutrients. 2022;14(13):2633. https://www.mdpi.com/2072-6643/14/13/2633. 35807814 10.3390/nu14132633 PMC9268456

[65] Baranwal N, Yu PK, Siegel NS. Sleep physiology, pathophysiology, and sleep hygiene. Prog Cardiovasc Dis. 2023;77:59–69. 10.1016/j.pcad.2023.02.005. 36841492 10.1016/j.pcad.2023.02.005

[66] Pires GN, Benedetto L, Cortese R, Gozal D, Gulia KK, Kumar VM, et al. Effects of sleep modulation during pregnancy in the mother and offspring: evidences from preclinical research. J Sleep Res. 2021;30(3):e13135. 10.1111/jsr.13135. 32618040 10.1111/jsr.13135

[67] Haufe A, Leeners B. Sleep Disturbances Across a Woman’s Lifespan: What Is the Role of Reproductive Hormones? J Endocr Soc. 2023;7(5). 10.1210/jendso/bvad036. 10.1210/jendso/bvad036 PMC10117379 37091307

[68] Beroukhim G, Esencan E, Seifer DB. Impact of sleep patterns upon female neuroendocrinology and reproductive outcomes: a comprehensive review. Reproductive Biology and Endocrinology. 2022;20(1):16.10.1186/s12958-022-00889-3. 10.1186/s12958-022-00889-3 PMC8764829 35042515

[69] Khanijow V, Prakash P, Emsellem HA, Borum ML, Doman DB. Sleep dysfunction and gastrointestinal diseases. Gastroenterol Hepatol. 2015;11(12):817–25. PMC4849511 27134599

[70] Li P, Wang H, Chen G, Feng J, Fan D, Lin D et al. Association between nausea and vomiting during pregnancy and sleep quality: mediating effect of depressive symptoms. Int J Gen Med. 2021:41–9. 10.2147/IJGM.S290216 PMC7802779 33447075

[71] Mislu E, Assalfew B, Arage MW, Chane F, Hailu T, Tenaw LA et al. Prevalence and factors associated with restless legs syndrome among pregnant women in middle-income countries: a systematic review and meta-analysis. Frontiers in medicine. 2023;10:1326337.10.3389/fmed.2023.1326337. 10.3389/fmed.2023.1326337 PMC10771314 38188334

[72] Fu T, Wang C, Yan J, Zeng Q, Ma C. Relationship between antenatal sleep quality and depression in perinatal women: a comprehensive meta-analysis of observational studies. J Affect Disord. 2023;327:38–45. 10.1016/j.jad.2023.01.125. 36739002 10.1016/j.jad.2023.01.125

[73] Manková D, Švancarová S, Štenclová E. Sleep, depression, anxiety, stress and circadian preferences among women in the third trimester. Are these variables related to mother’s expectations about their child’s sleep? Curr Psychol. 2024;43(22):19985–95. 10.1007/s12144-024-05839-3.

[74] Mislu E, Kumsa H, Arage MW, Tadesse S, Chane F. Prevalence of poor sleep quality among pregnant women in low- and middle-income countries: A systematic review and meta-analysis. American Journal of Obstetrics & Gynecology MFM.10.1016/j.ajogmf.2024.101381. 10.1016/j.ajogmf.2024.101381 38759871

[75] Baron KG, Duffecy J, Reutrakul S, Levenson JC, McFarland MM, Lee S, et al. Behavioral interventions to extend sleep duration: a systematic review and meta-analysis. Sleep Med Rev. 2021;60:101532. 10.1016/j.smrv.2021.101532. 34507028 10.1016/j.smrv.2021.101532 PMC10476177

