# Supplementary material for: Sleep quality disparities in different pregnancy trimesters in low- and middle-income countries: a systematic review and meta-analysis
Source: BMC Pregnancy Childbirth. 2024 Oct 1;24:627. doi: 10.1186/s12884-024-06830-3 (PMC11446071; doi:10.1186/s12884-024-06830-3)
Supplement: Supplementary file 1 — Supplementary Material 1 [file 12884_2024_6830_MOESM1_ESM.docx]

Supplementary Figure 1. Funnel plot for included studies for poor quality of sleep among first trimester pregnant women in low and middle-income countries

Supplementary Figure 2. Funnel plot for poor quality of sleep among pregnant women during the second trimester of pregnancy in low and middle-income countries

**
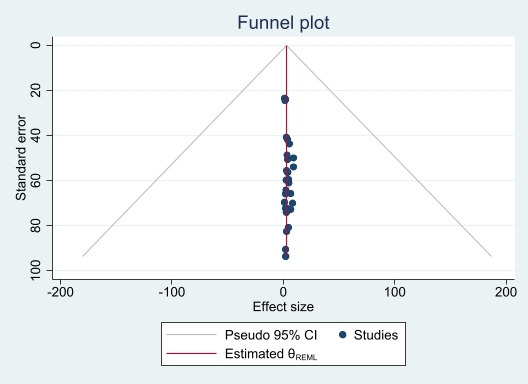
**

Supplementary Figure 3. Funnel plot for poor quality of sleep among third trimester pregnant women in low and middle-income countries
