# Supplementary material for: Sleep quality disparities in different pregnancy trimesters in low- and middle-income countries: a systematic review and meta-analysis
Source: BMC Pregnancy Childbirth. 2024 Oct 1;24:627. doi: 10.1186/s12884-024-06830-3 (PMC11446071; doi:10.1186/s12884-024-06830-3)
Supplement: Supplementary file 2 — Supplementary Material 2 [file 12884_2024_6830_MOESM2_ESM.docx]

| **Supplementary Table 1. Overview of Study Characteristics in the Meta-Analysis to Evaluating Discrepancies in Poor Sleep Quality by Trimester of Pregnancy** | | | | | | | | | | | |
| --- | --- | --- | --- | --- | --- | --- | --- | --- | --- | --- | --- |
| **Authors** | **Year** | **Country** | **Title of the study** | **Income Category based on World Bank** | **Study design** | **Study setting** | **Study Population** | **Sample Size** | **First Trimester prevalence** | **Second Trimester prevalence** | **Third Trimester prevalence** |
| Natália Amorim et al | 2023 | Brazil | Sleep in pregnancy quarters: a longitudinal study | Upper middle income | Cross-sectional | Institution based | ANC follow up | 50 | 55.3 | 39.5 | 65.8 |
| Huishan Zhang et al | 2021 | China | Prevalence of and Risk Factors for Poor Sleep During Different Trimesters of Pregnancy Among Women in China: A Cross-Sectional Study | Upper middle income | Cross-sectional | Institution based | Pregnant women | 2281 | 54.3 | 49.3 | 69.6 |
| Min Du et al | 2020 | China | Maternal sleep quality during early pregnancy, risk factors and its impact on pregnancy outcomes: a prospective cohort study | Upper middle income | Cross-sectional | Institution based | ANC follow up | 4352 | 34.14 |  |  |
| Shao-Yu Tsai et al | 2011 | China | Factors Associated With Sleep Quality in Pregnant Women | Upper middle income | Cross-sectional | Institution based | third trimester pregnancy | 30 |  |  | 50 |
| Guopeng Li et al | 2016 | China | Relationship between prenatal maternal stress and sleep quality in Chinese pregnant women: the mediation effect of resilience | Low income | Cross-sectional | Institution based | second TM pregnant women | 227 |  | 38.8 |  |
| Yunhan Yu et al | 2020 | China | of depression symptoms and its influencing factors among pregnant women in late pregnancy in urban areas of Hengyang City, Hunan Province, China: a … | Upper middle income | Cross-sectional | Institution based | uncomplicated pregnancy | 813 |  |  | 24.6 |
| Yi-Min Cai et al | 2018 | China | Study on the sleep quality of women pregnant with a second child and the influencing factors | Upper middle income | Cross-sectional | Institution based | pregnant with a second child | 162 | 27.27 | 30.43 | 41.9 |
| Nan Feng et al | 2020 | China | anxiety, sleep disorders and influencing factors among pregnant women in the second trimester in urban areas of Guangzhou city, Guangdong province, China … | Upper middle income | Cross-sectional | Institution based | pregnant women | 553 |  | 31.1 |  |
| Ying Sun et al | 2023 | China | Risk factors of positive depression screening during the third trimester of pregnancy in a Chinese tertiary hospital: a cross-sectional study | Upper middle income | Cross-sectional | Institution based | third trimester pregnancy | 1196 |  |  | 23.49 |
| Shirong Cai et al | 2016 | China | Mid‐pregnancy and postpartum maternal mental health and infant sleep in the first year of life | Upper middle income | Cross-sectional | Institution based | second TM pregnant women | 686 |  | 43.1 |  |
| Shao-Yu Tsai et al | 2016 | China | Cross-sectional and longitudinal associations between sleep and health-related quality of life in pregnant women: A prospective observational study | Upper middle income | Cross-sectional | Institution based | pregnant women | 164 | 43.3 | 37.2 | 50.6 |
| Yi-Li Ko et al | 2015 | China | Stress, sleep quality and unplanned C aesarean section in pregnant women | Upper middle income | Cross-sectional | Institution based | early stage labor | 200 |  |  | 90.5 |
| Aman Dule et al | 2020 | Ethiopia | Sleep Quality Among Pregnant Women Amidst COVID-19: Association with Mental Wellbeing and Self-efficacy | Low income | Cross-sectional | Institution based | ANC follow up | 228 | 63.1 | 58.8 | 48.7 |
| [Nakachew S. et al](https://www.frontiersin.org/people/u/1546676) | 2020 | Ethiopia | Determinants of Poor Sleep Quality During the COVID-19 Pandemic Among Women Attending Antenatal Care Services at the Health Facilities of Debre Berhan Town, Ethiopia: An Institutional-Based Cross-Sectional Study | Low income | Cross-sectional | Institution based | ANC follow up | 423 | 42.9 | 55.8 | 72.2 |
| Girmaw M. et al | 2022 | Ethiopia | Sleep quality and associated factors among pregnant women attending antenatal care unit at Gondar, Ethiopia: a cross-sectional study | Low income | Cross-sectional | Institution based | ANC follow up | 415 | 16 | 60 | 24 |
| [Abdulhenan A. et al](https://www.tandfonline.com/author/Abdurahman%2C+Abdulhenan) | 2021 | Ethiopia | Sleep Quality and Associated Factors among Pregnant Women Attending Antenatal Care Unit at the Referral Hospitals in Oromia National Regional State, Ethiopia | Low income | Cross-sectional | Institution based | ANC follow up | 414 | 44.8 | 36.8 | 64.2 |
| Hayat Tuha | 2021 | Ethiopia | Quality of Sleep and Associated Factors among Pregnant Women in AddisAbaba Public Hospitals | Low income | Cross-sectional | Institution based | pregnant women | 404 | 11.76 | 28.43 | 59.8 |
| Kranti S. Kadam et al | 2023 | India | Depression, sleep quality, and body image disturbances among pregnant women in India: a cross-sectional study | Lower middle income | Cross-sectional | Institution based | out patient obstetric and gynecology unit | 146 | 38.8 | 44.9 | 56.3 |
| Abhishek Ghante et al | 2021 | India | Prevalence and predictors of sleep deprivation and poor sleep quality and their associated perinatal outcomes during the third trimester of pregnancy | Lower middle income | Cross-sectional | Institution based | delivery unit at discharge | 225 |  |  | 72.9 |
| Yuni Astuti et al | 2019 | Indonesia | The Factors Influencing Sleep Quality of Pregnant Women in Yogyakarta, Indonesia | Upper middle income | Cross-sectional | Institution based | No complication during pregnancy ANC | 161 | 80 | 75 | 82.5 |
| Dian Rahmawati et al | 2017 | Indonesia | Anxiety, Depression, and Sleep Quality of Third Trimester Pregnant Women | Upper middle income | Cross-sectional | Institution based | third trimester pregnancy | 110 |  |  | 93.6 |
| Anisah Tifani M. et al | 2019 | Indonesia | The Relationship Between Sleep Quality And Depression During The Third Trimester Of Pregnant Women In The Public Health Center In Bengkulu City | Upper middle income | Cross-sectional | Institution based | third trimester pregnancy | 95 |  |  | 61.1 |
| Hilaliah et al | 2021 | Indonesia | Relationship Of Obesity, Anxiety Level And Sleep Quality In Pregnant Women With Preeclamsia In Rsud Dr. Hm Rabain Muara Enim 2021 | Upper middle income | Cross-sectional | Institution based | greater than 20 weeks | 46 |  | 56.5 |  |
| Ulty Desmarnita et al | 2020 | Indonesia | Discomfort of pregnant women on the quality sleep of third trimester pregnant women | Upper middle income | Cross-sectional | Institution based | third trimester pregnancy | 30 |  |  | 70 |
| Rahmi A. et al | 2022 | Indonesia | The Association of Maternal Sleep Quality with Newborn Health | Upper middle income | Cross-sectional | Institution based | term pregnant women | 78 |  |  | 80.8 |
| Koochaksaraei FY et al | 2022 | Iran | Late-pregnancy sleep quality and psychological distress in Iranian primiparous women | Lower middle income | Cross-sectional | Institution based | Primiparous | 300 |  |  | 74 |
| Abolfazl M. et al | 2019 | Iran | The Frequency of Pregnancy Associated Sleep Disorders Among Pregnant Mothers Who Referred to Rasoul-Akram Hospital for Prenatal Care 2018 - 2019 | Lower middle income | Cross-sectional | Institution based | referred pregnant women | 343 |  | 46.9 | 40.8 |
| I. Naghi et al | 2011 | Iran | Sleep disturbance in late pregnancy and type and duration of labour | Lower middle income | Cross-sectional | Institution based | third trimester pregnancy | 88 |  |  | 43.8 |
| Ai Ni Teoh et al | 2021 | Malaysia | Psychological state during pregnancy is associated with sleep quality: preliminary findings from MY-CARE cohort study | Upper middle income | Cross-sectional | Institution based | second TM pregnant women | 179 |  | 61.8 |  |
| Zubair UB et al | 2016 | Pakistan | Assessment of Quality of Sleep in Pregnancy and Socio Demographic Factors Associated with Poor Sleep Quality in Pregnancy | Lower middle income | Cross-sectional | Institution based | ANC follow up | 114 | 27.4 |  | 72.6 |
| Naseem Ahmed et al | 2019 | Pakistan | Prevalence of sleep disturbances during pregnancy–a pilot study | Lower middle income | Cross-sectional | Institution based | visiting obs and gyne | 30 |  | 60 | 54 |
| Bizu Gelaye et al | 2014 | Peru | Poor sleep quality, antepartum depression and suicidal ideation among pregnant women | Upper middle income | Cross-sectional | Institution based | second TM pregnant women | 1298 | 17 |  |  |
| Sixto E. Sanchez et al | 2016 | Peru | Intimate partner violence is associated with stress-related sleep disturbance and poor sleep quality during early pregnancy | Upper middle income | Cross-sectional | Institution based | pregnant women | 634 | 28.07 |  |  |
| Samoilova Y.S et al | 2023 | Russia | Screening for sleep apnea, daytime sleepiness, sleep quality, anxiety and depressive disorders in pregnant women in the third trimester of pregnancy | Upper middle income | Cross-sectional | Institution based | third trimester pregnancy | 44 |  |  | 72.727 |
| D Yasaratne et al | 2021 | Sri Lanka | Factors associated with poor sleep quality and excessive daytime sleepiness in late pregnancy: A pilot study in an antenatal unit | Lower middle income | Cross-sectional | Institution based | third trimester pregnancy | 109 |  |  | 59.6 |
| Thuvachit K. et al | 2018 | Thailand | Brain-Derived Neurotrophic Factor (BDNF) Depression and Subjective Sleep Quality in the First Trimester of Pregnancy Among Migrant Workers in Thailand | Upper middle income | Cross-sectional | Institution based | first trimester pregnancy | 108 | 28.7 |  |  |
| Sanem Nemmezi K. et al | 2022 | Turkey | Evaluation of sleep quality and related factors in pregnant women | Upper middle income | Cross-sectional | Institution based | Pregnant women who applied to ob-gyn clinic for outpatient follow up | 256 | 42.9 | 50 | 55.7 |
| İlknur Dolu et al | 2020 | Turkey | Factors affecting subjective sleep quality in the third trimester of pregnancy in urban areas of Turkey and Iraq | Upper middle income | Cross-sectional | Institution based | third trimester pregnancy | 582 |  |  | 65.979 |
